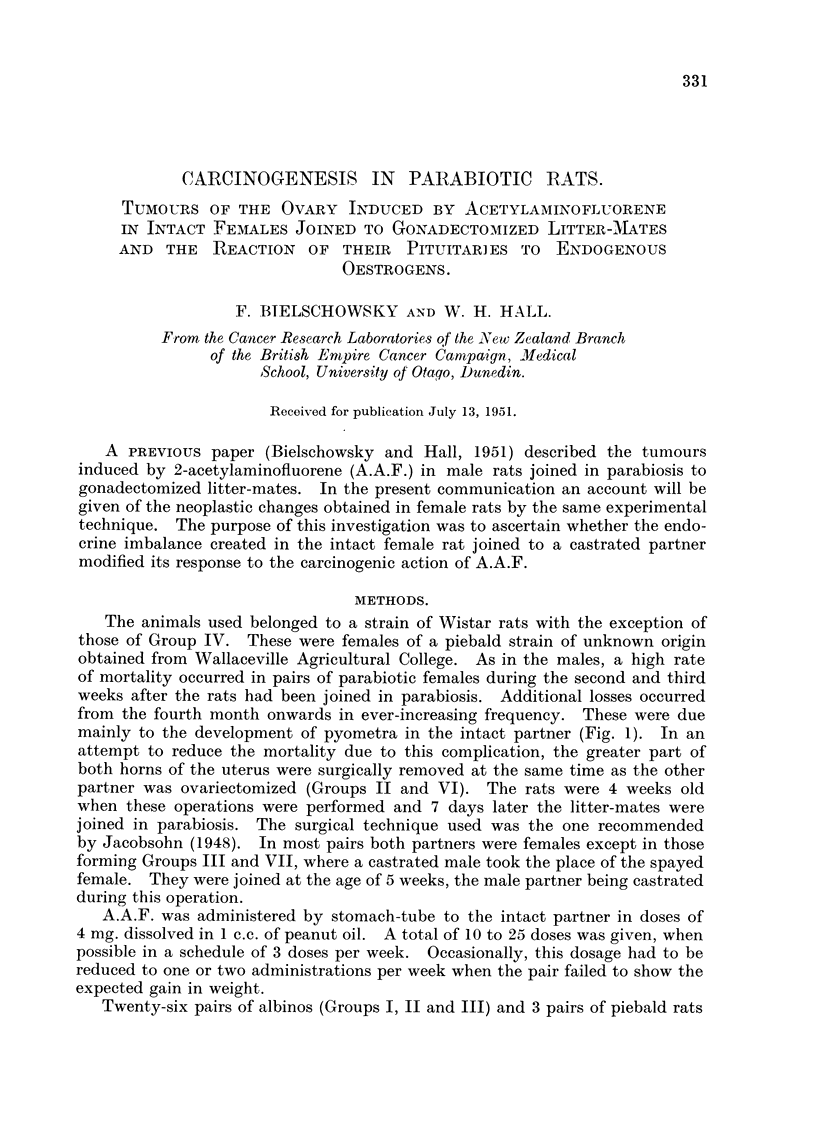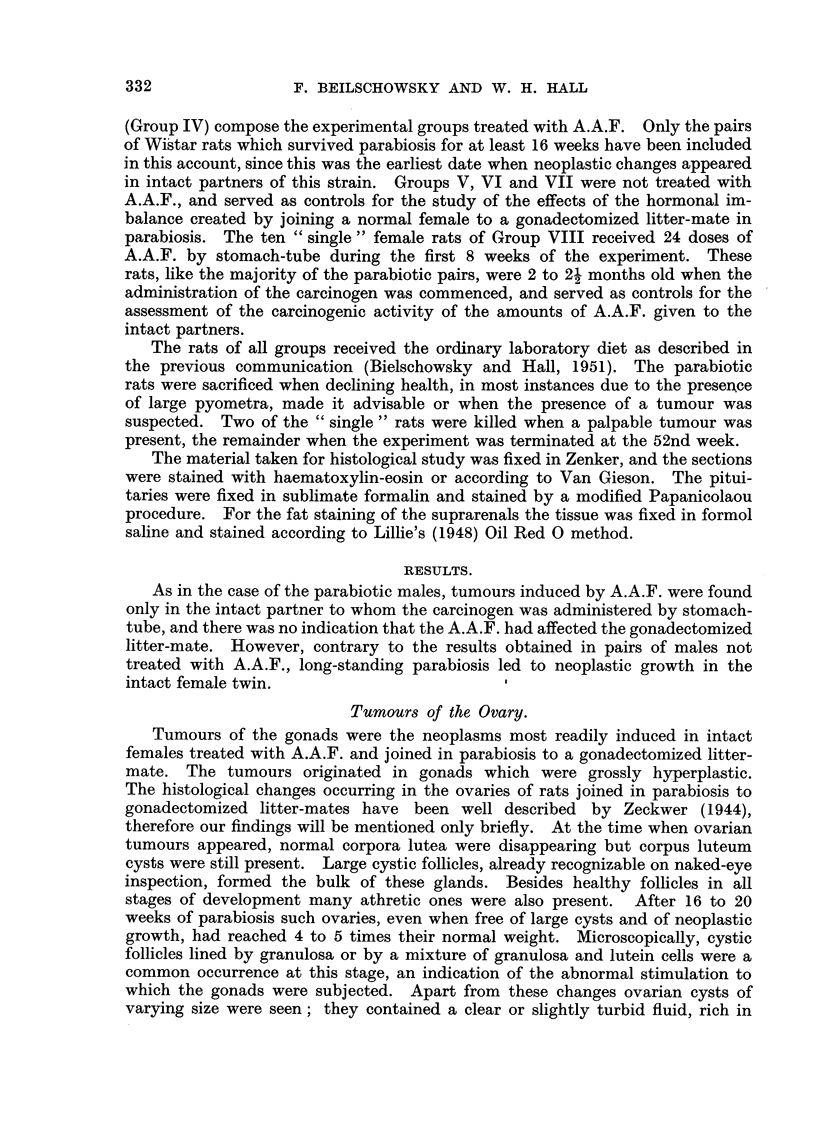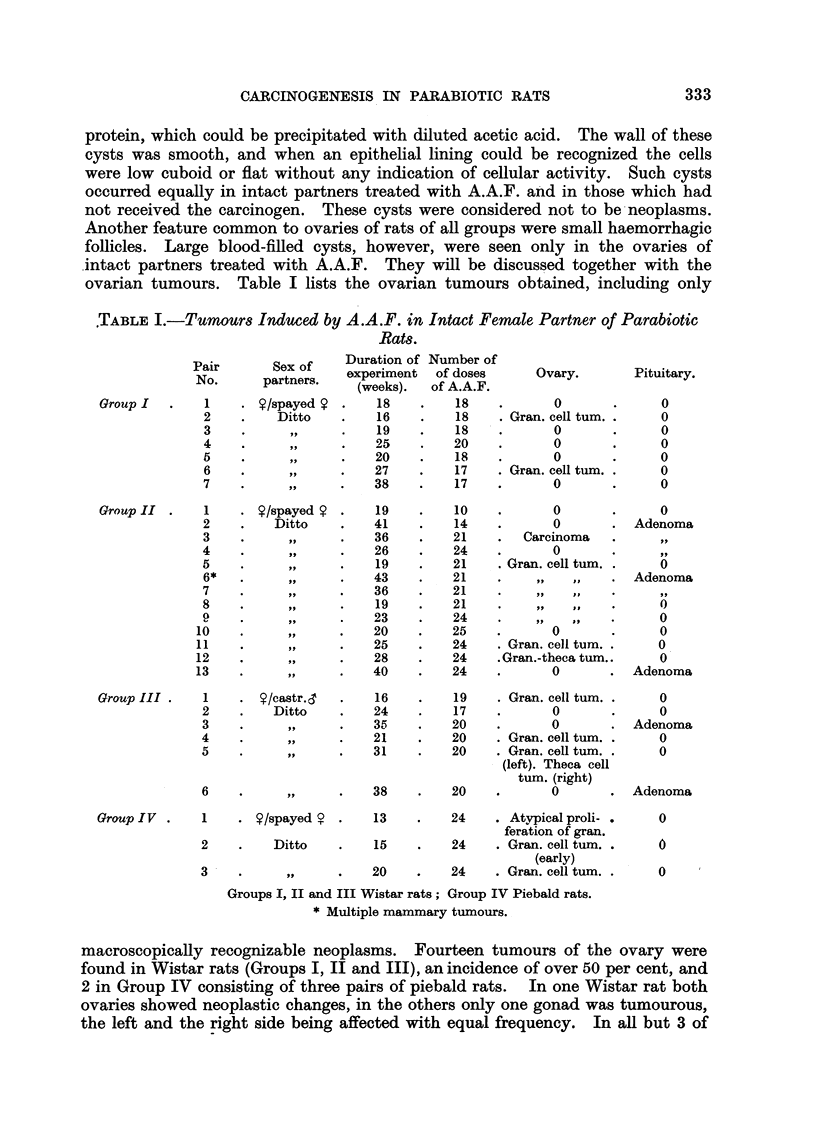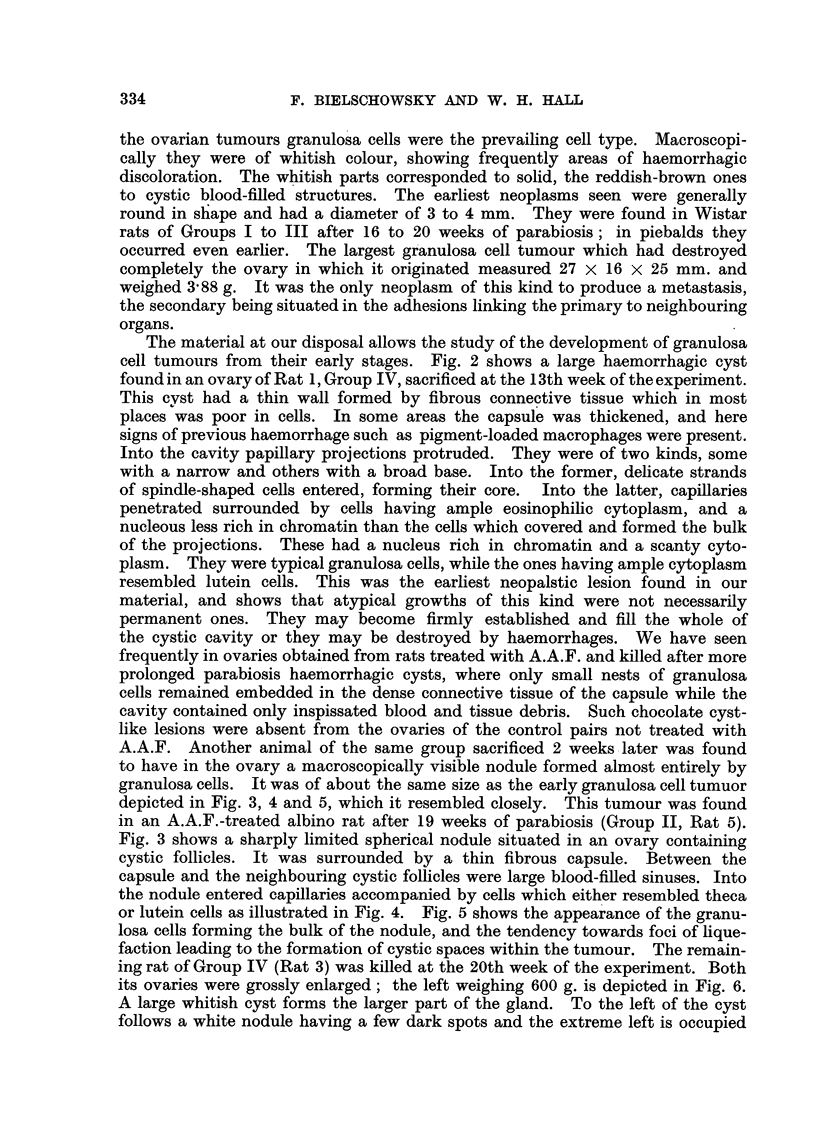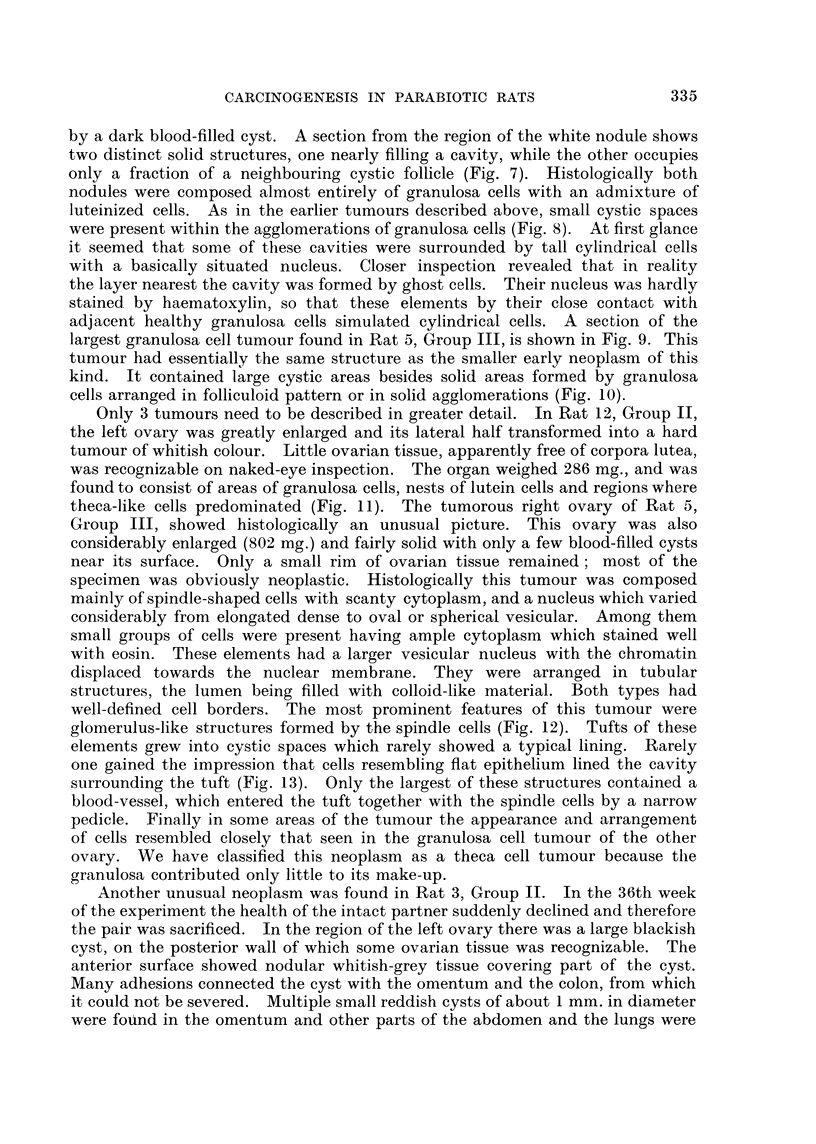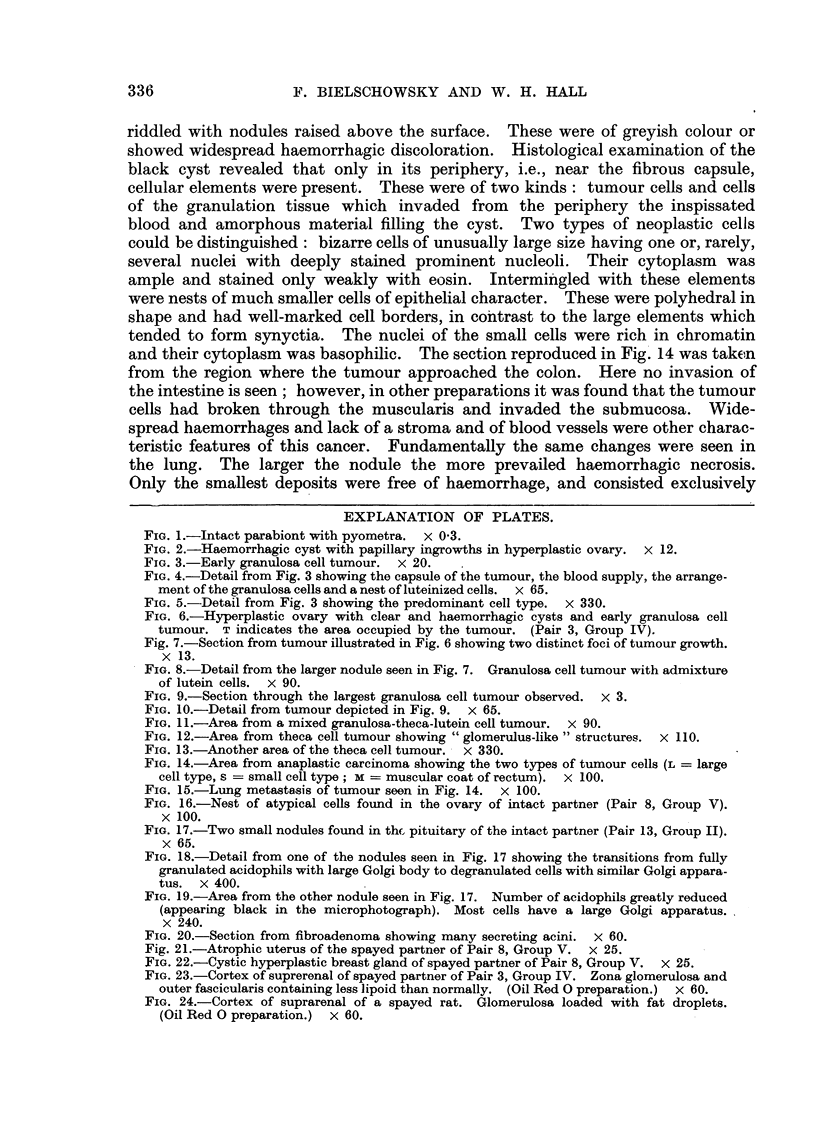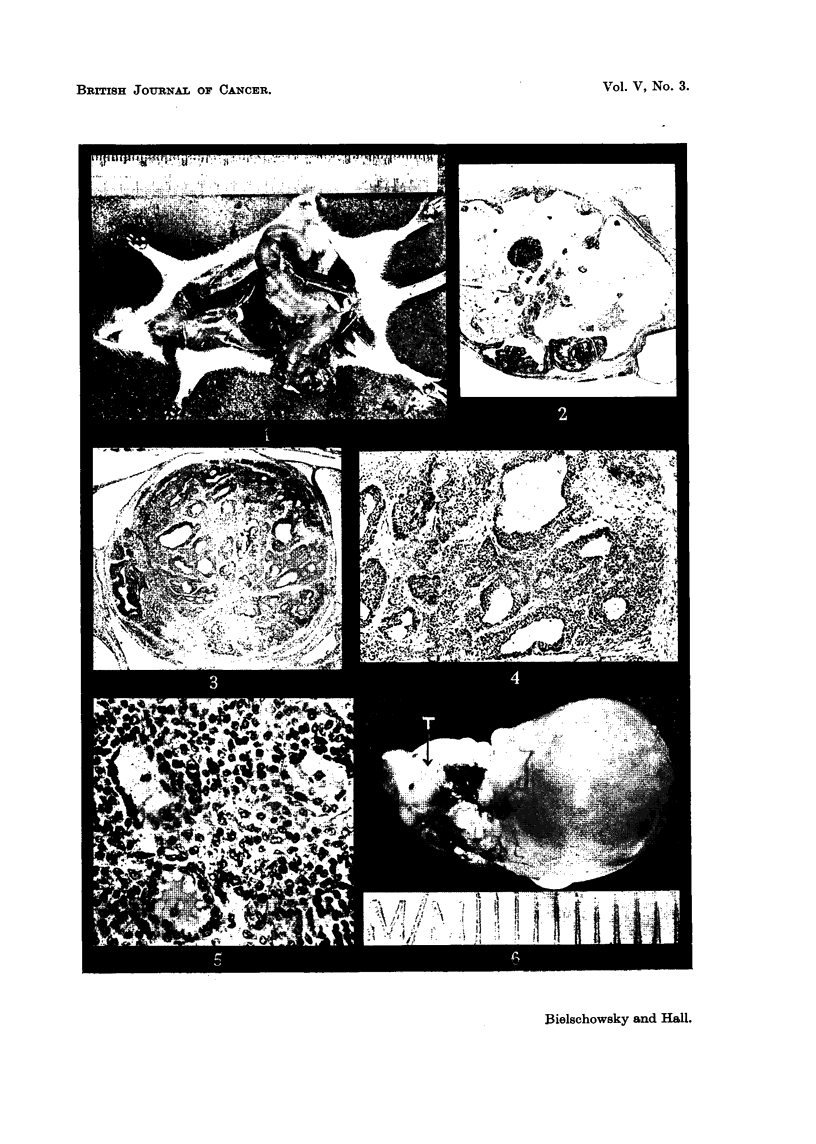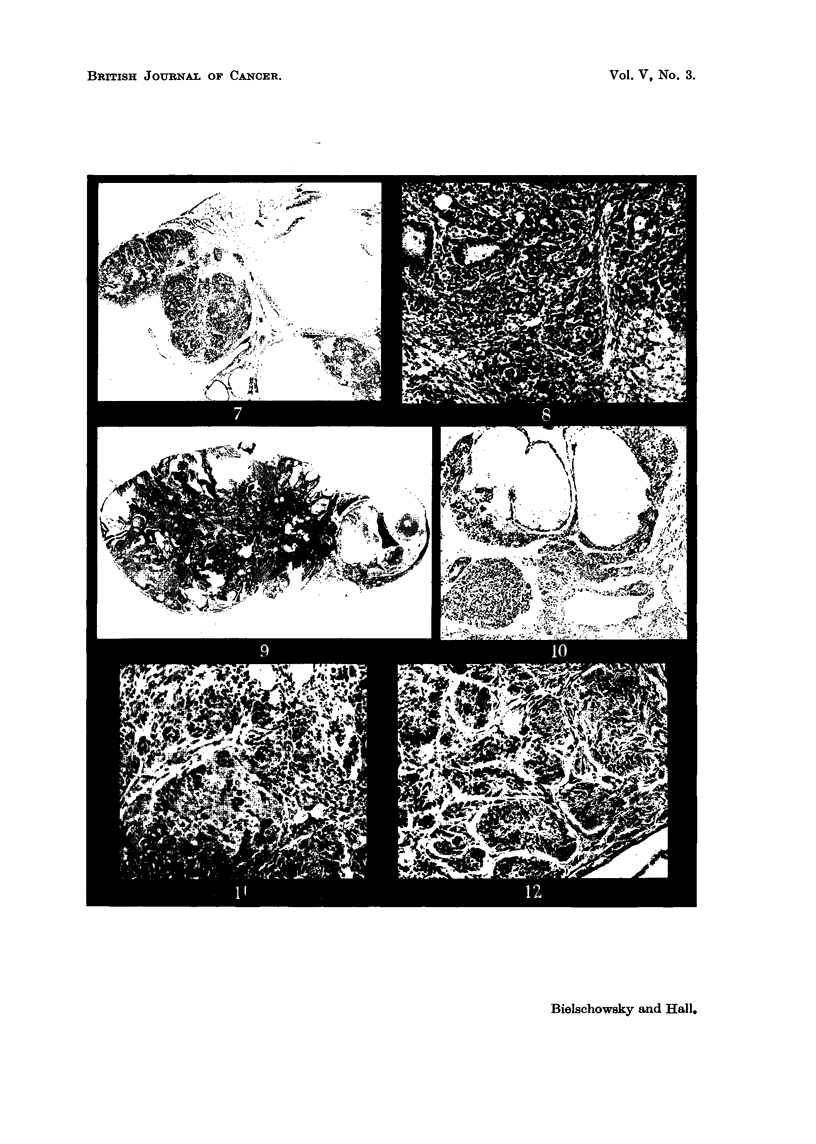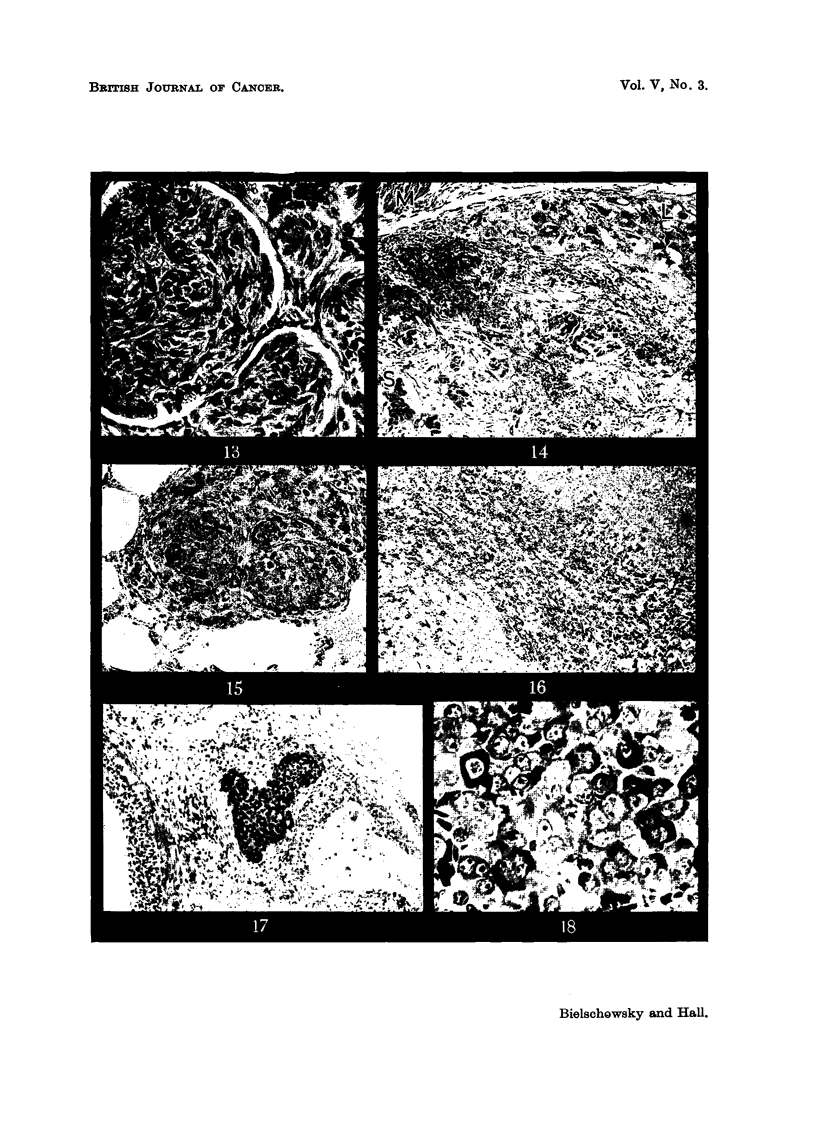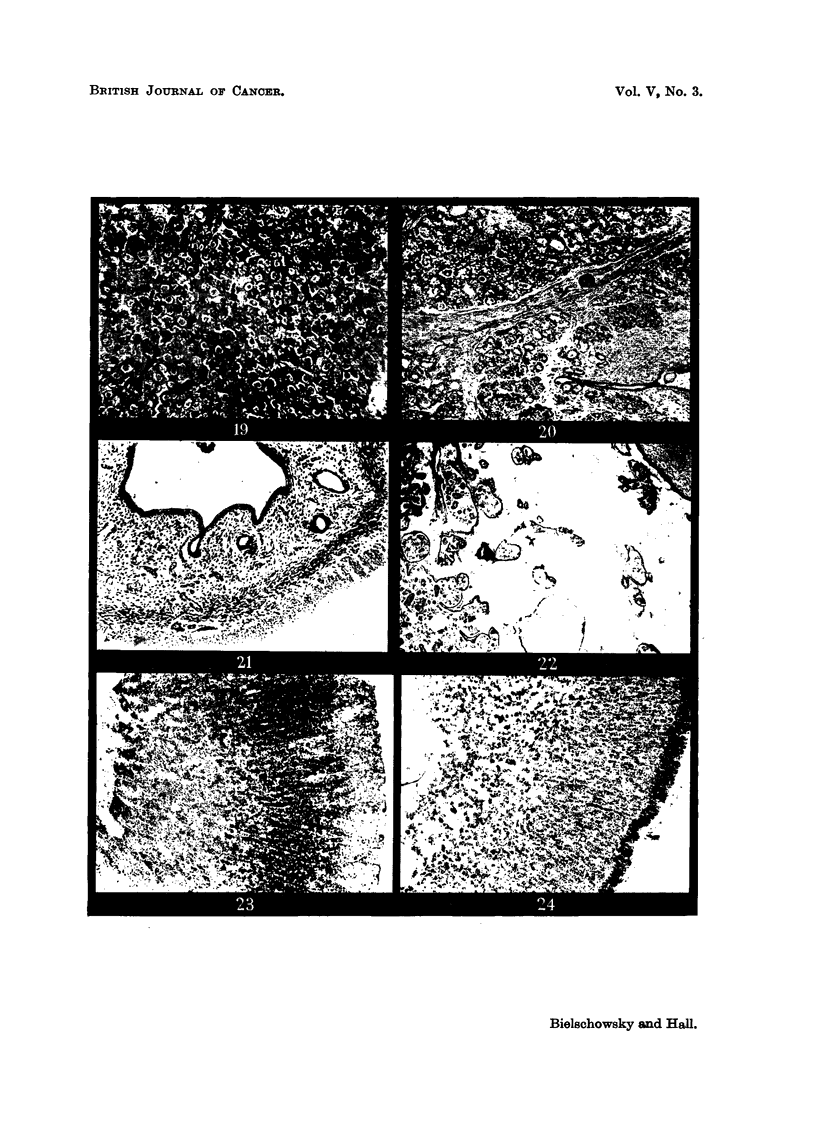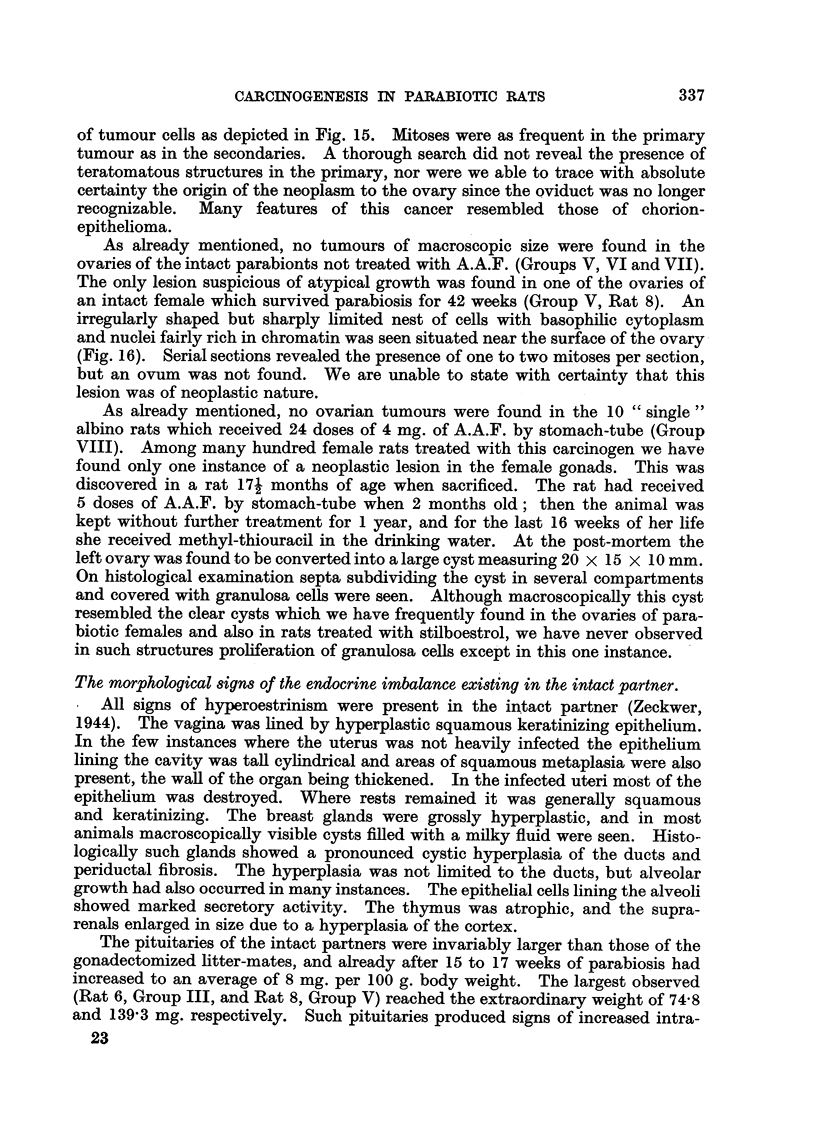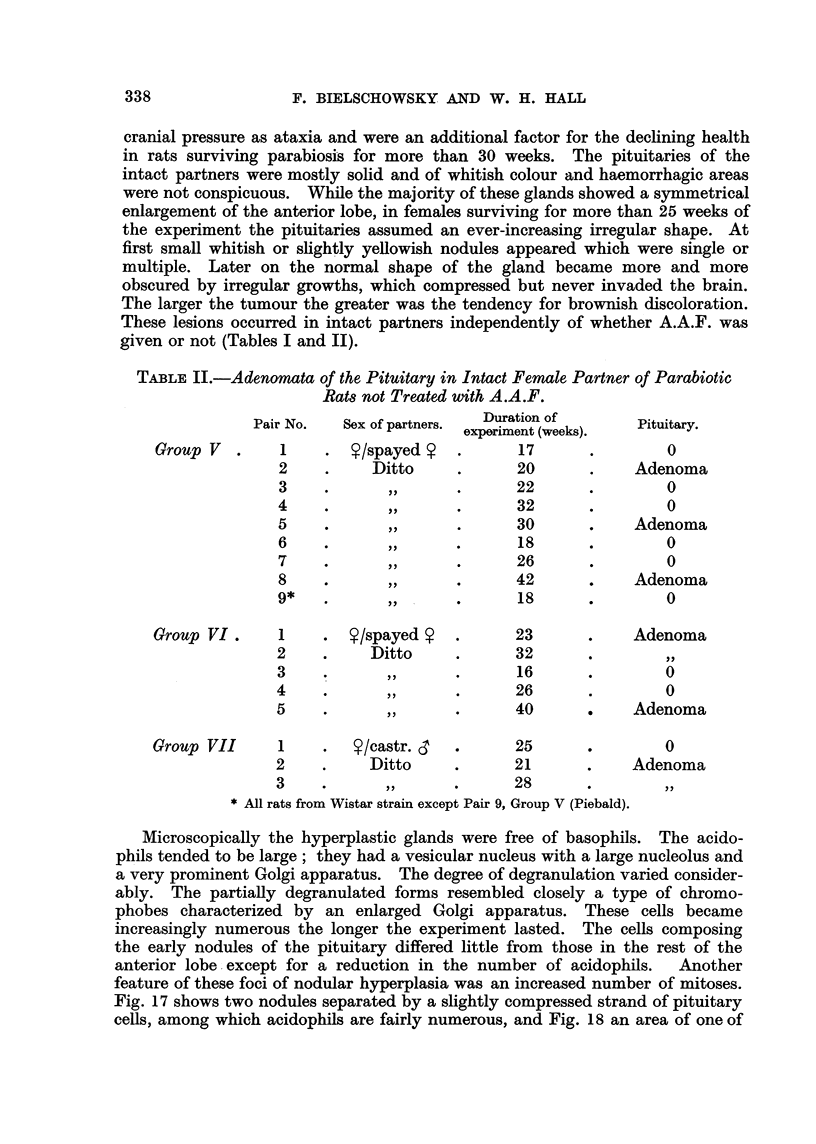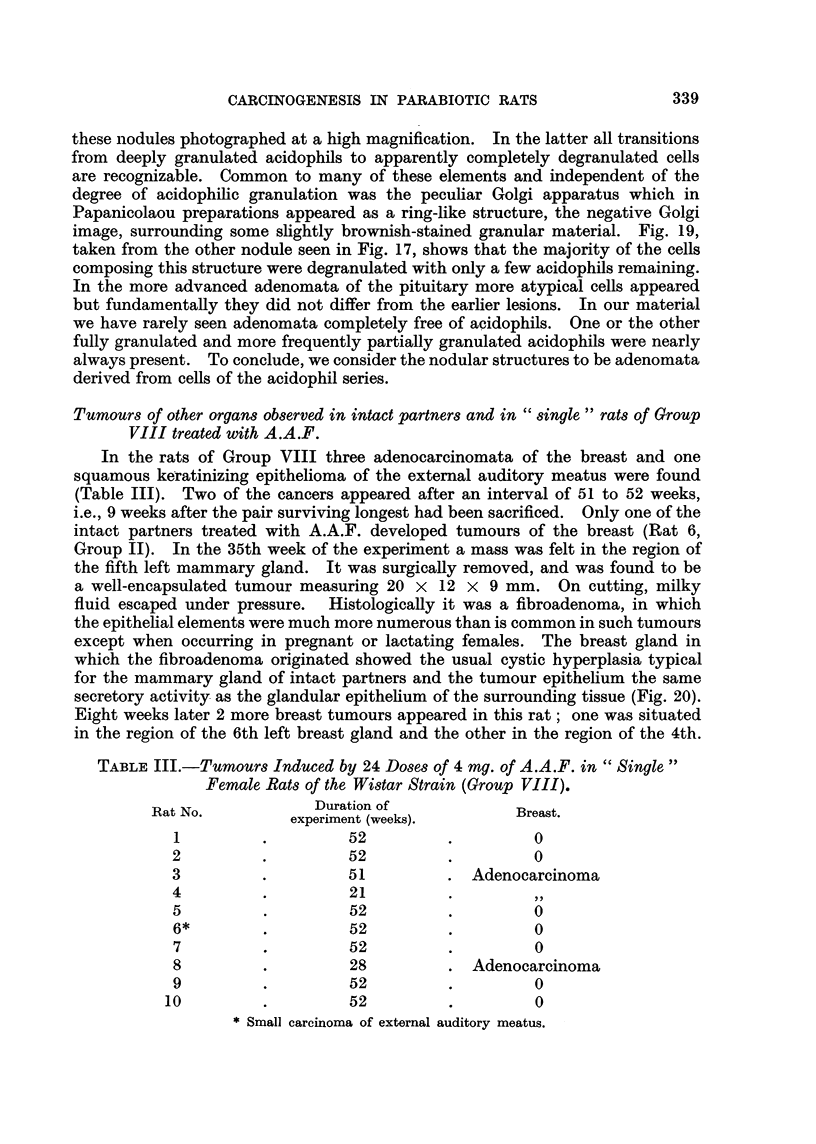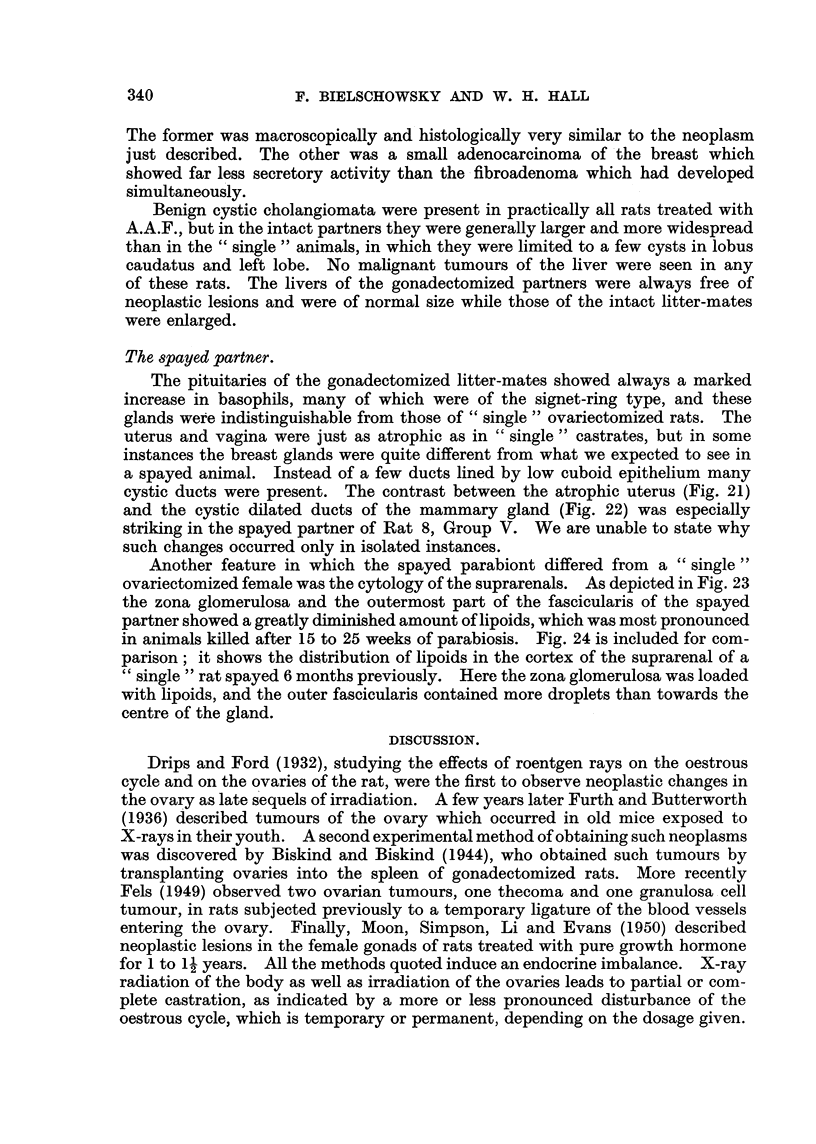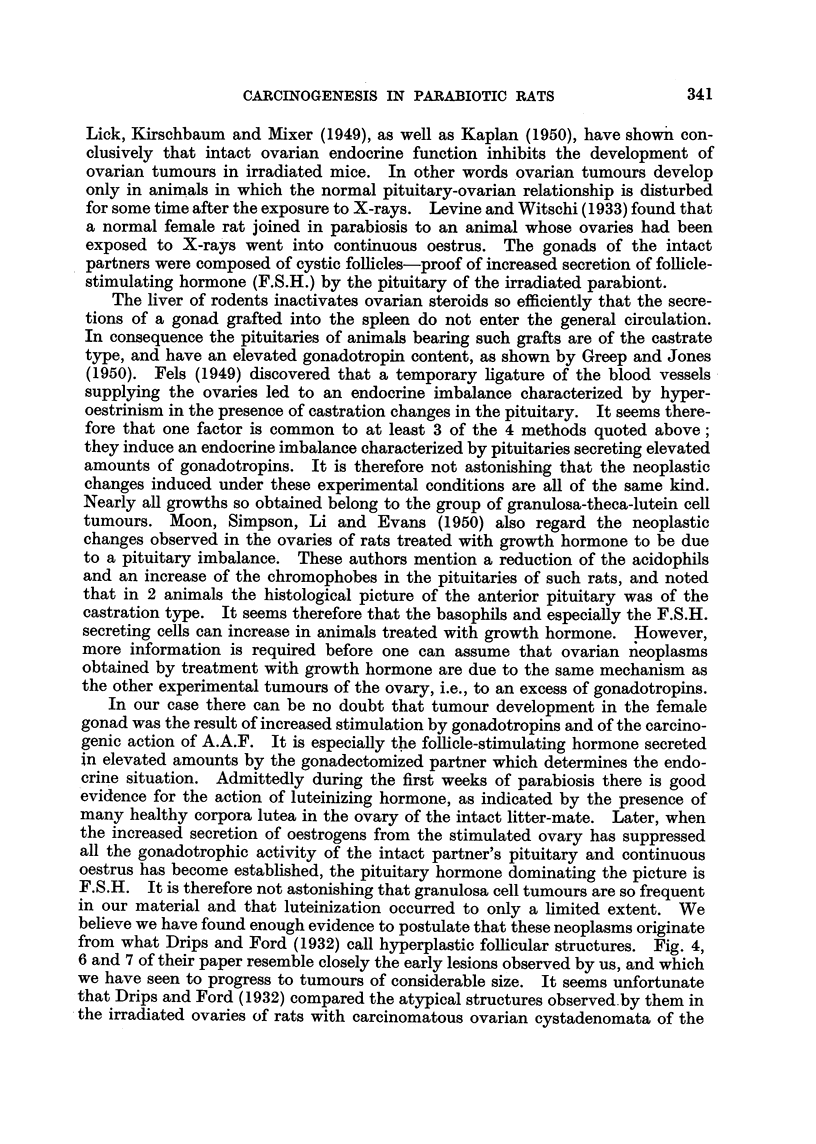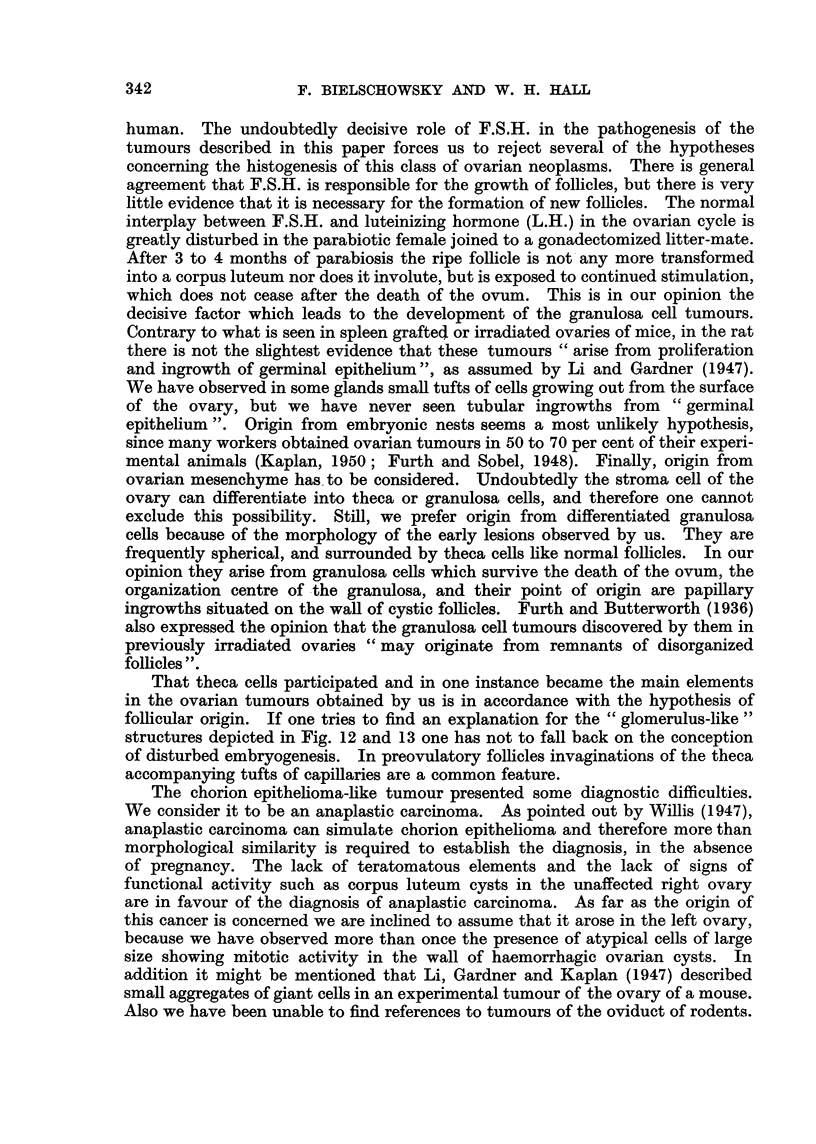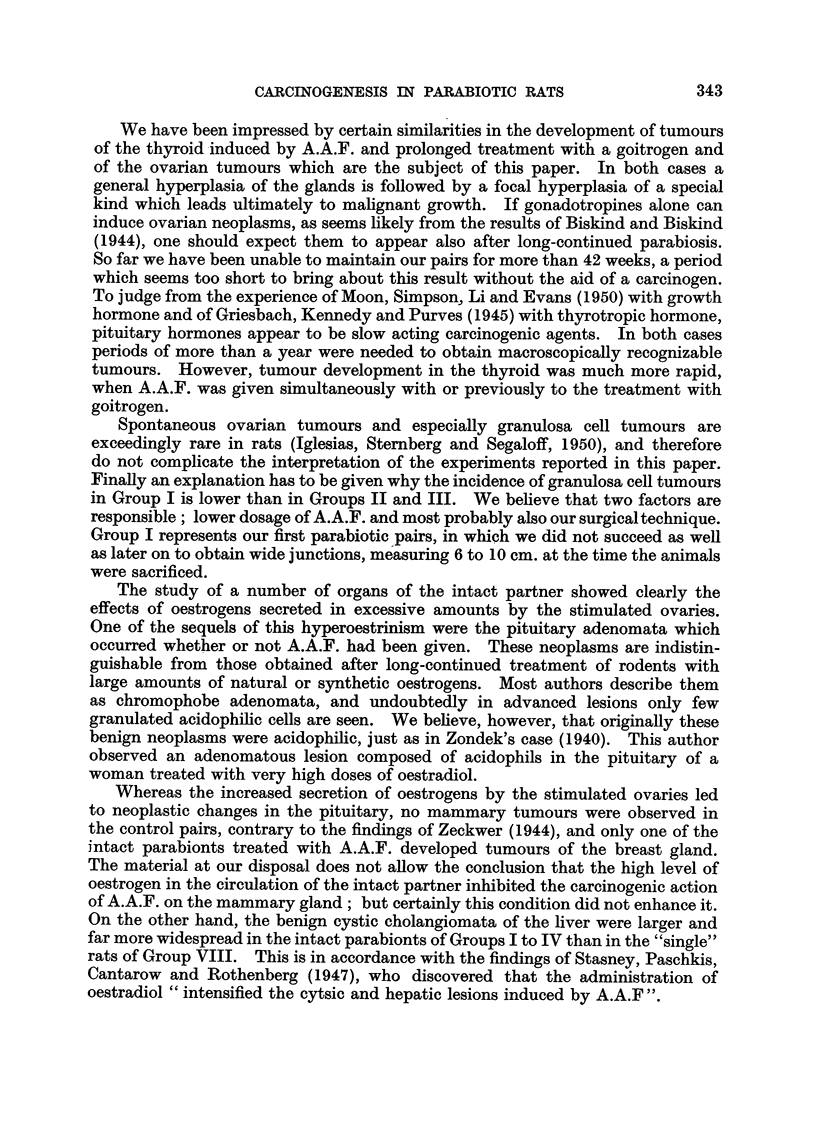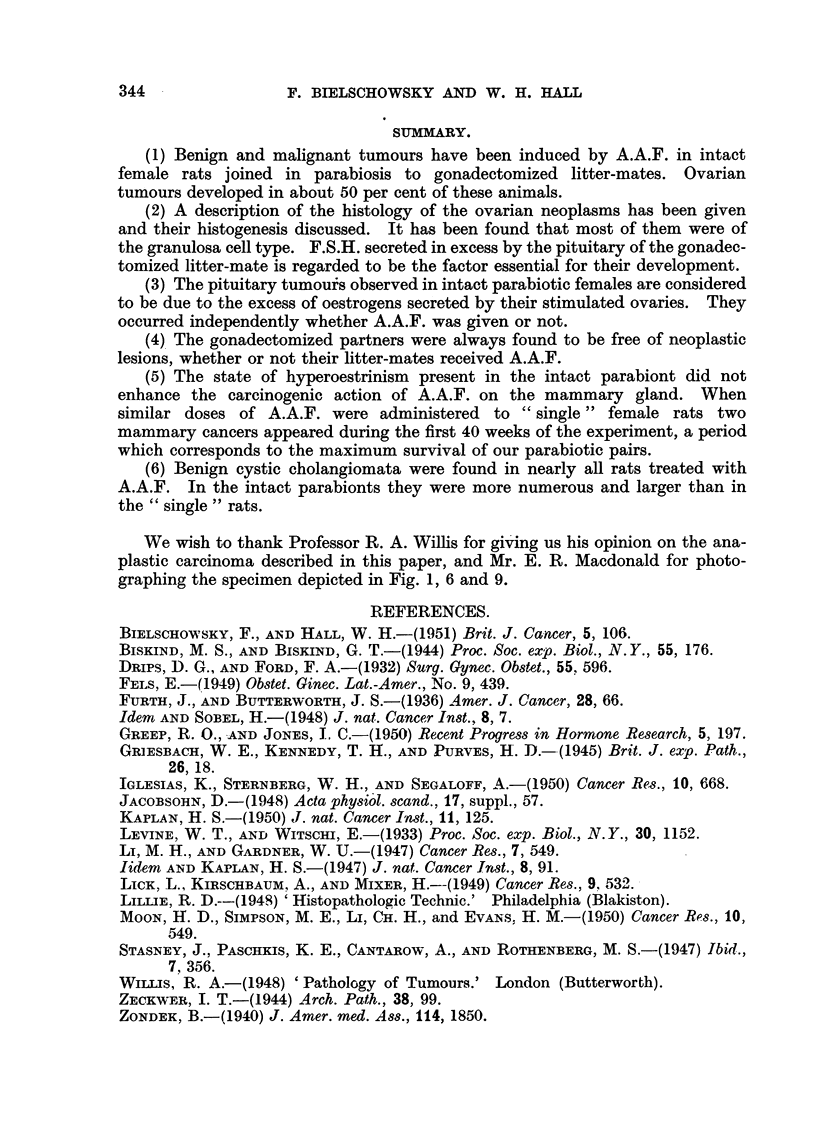# Carcinogenesis in Parabiotic Rats

**DOI:** 10.1038/bjc.1951.35

**Published:** 1951-09

**Authors:** F. Bielschowsky, W. H. Hall

## Abstract

**Images:**


### CARCINOGENESIS IN PARABIOTIC RATS.

### TUMOURS OF THE OVARY INDUCED BY ACETYLAMINOFLUORENE IN INTACT FEMALES JOINED TO GONADECTOMIZED LITTER-MATES AND THE REACTION OF THEIR PITUITARIES TO ENDOGENOUS OESTROGENS.

F. BIELSCHOWSKY AND W. H. HALL.

*From the Cancer Research Laboratories of the New Zealand Branch of the British Empire Cancer Campaign, Medical School, University of Otago, Dunedin.*

Received for publication July 13, 1951.

A PREVIOUS paper (Bielschowsky and Hall, 1951) described the tumours induced by 2-acetylaminofluorene (A.A.F.) in male rats joined in parabiosis to gonadectomized litter-mates. In the present communication an account will be given of the neoplastic changes obtained in female rats by the same experimental technique. The purpose of this investigation was to ascertain whether the endocrine imbalance created in the intact female rat joined to a castrated partner modified its response to the carcinogenic action of A.A.F.

### METHODS.

The animals used belonged to a strain of Wistar rats with the exception of those of Group IV. These were females of a piebald strain of unknown origin obtained from Wallaceville Agricultural College. As in the males, a high rate of mortality occurred in pairs of parabiotic females during the second and third weeks after the rats had been joined in parabiosis. Additional losses occurred from the fourth month onwards in ever-increasing frequency. These were due mainly to the development of pyometra in the intact partner (Fig. 1). In an attempt to reduce the mortality due to this complication, the greater part of both horns of the uterus were surgically removed at the same time as the other partner was ovariectomized (Groups II and VI). The rats were 4 weeks old when these operations were performed and 7 days later the litter-mates were joined in parabiosis. The surgical technique used was the one recommended by Jacobsohn (1948). In most pairs both partners were females except in those forming Groups III and VII, where a castrated male took the place of the spayed female. They were joined at the age of 5 weeks, the male partner being castrated during this operation.

A.A.F. was administered by stomach-tube to the intact partner in doses of 4 mg. dissolved in 1 c.c. of peanut oil. A total of 10 to 25 doses was given, when possible in a schedule of 3 doses per week. Occasionally, this dosage had to be reduced to one or two administrations per week when the pair failed to show the expected gain in weight.

Twenty-six pairs of albinos (Groups I, II and III) and 3 pairs of piebald rats

(Group IV) compose the experimental groups treated with A.A.F. Only the pairs of Wistar rats which survived parabiosis for at least 16 weeks have been included in this account, since this was the earliest date when neoplastic changes appeared in intact partners of this strain. Groups V, VI and VII were not treated with A.A.F., and served as controls for the study of the effects of the hormonal imbalance created by joining a normal female to a gonadectomized litter-mate in parabiosis. The ten " single " female rats of Group VIII received 24 doses of A.A.F. by stomach-tube during the first 8 weeks of the experiment. These rats, like the majority of the parabiotic pairs, were 2 to 2½ months old when the administration of the carcinogen was commenced, and served as controls for the assessment of the carcinogenic activity of the amounts of A.A.F. given to the intact partners.

The rats of all groups received the ordinary laboratory diet as described in the previous communication (Bielschowsky and Hall, 1951). The parabiotic rats were sacrificed when declining health, in most instances due to the presence of large pyometra, made it advisable or when the presence of a tumour was suspected. Two of the " single " rats were killed when a palpable tumour was present, the remainder when the experiment was terminated at the 52nd week.

The material taken for histological study was fixed in Zenker, and the sections were stained with haematoxylin-eosin or according to Van Gieson. The pituitaries were fixed in sublimate formalin and stained by a modified Papanicolaou procedure. For the fat staining of the suprarenals the tissue was fixed in formol saline and stained according to Lillie's (1948) Oil Red O method.

### RESULTS.

As in the case of the parabiotic males, tumours induced by A.A.F. were found only in the intact partner to whom the carcinogen was administered by stomach-tube, and there was no indication that the A.A.F. had affected the gonadectomized litter-mate. However, contrary to the results obtained in pairs of males not treated with A.A.F., long-standing parabiosis led to neoplastic growth in the intact female twin.

### *Tumours of the Ovary.*

Tumours of the gonads were the neoplasms most readily induced in intact females treated with A.A.F. and joined in parabiosis to a gonadectomized litter-mate. The tumours originated in gonads which were grossly hyperplastic. The histological changes occurring in the ovaries of rats joined in parabiosis to gonadectomized litter-mates have been well described by Zeckwer (1944), therefore our findings will be mentioned only briefly. At the time when ovarian tumours appeared, normal corpora lutea were disappearing but corpus luteum cysts were still present. Large cystic follicles, already recognizable on naked-eye inspection, formed the bulk of these glands. Besides healthy follicles in all stages of development many athretic ones were also present. After 16 to 20 weeks of parabiosis such ovaries, even when free of large cysts and of neoplastic growth, had reached 4 to 5 times their normal weight. Microscopically, cystic follicles lined by granulosa or by a mixture of granulosa and lutein cells were a common occurrence at this stage, an indication of the abnormal stimulation to which the gonads were subjected. Apart from these changes ovarian cysts of varying size were seen ; they contained a clear or slightly turbid fluid, rich in protein, which could be precipitated with diluted acetic acid. The wall of these cysts was smooth, and when an epithelial lining could be recognized the cells were low cuboid or flat without any indication of cellular activity. Such cysts occurred equally in intact partners treated with A.A.F. and in those which had not received the carcinogen. These cysts were considered not to be neoplasms. Another feature common to ovaries of rats of all groups were small haemorrhagic follicles. Large blood-filled cysts, however, were seen only in the ovaries of intact partners treated with A.A.F. They will be discussed together with the ovarian tumours. Table I lists the ovarian tumours obtained, including only

TABLE I.—*Tumours Induced by A.A.F. in Intact Female Partner of Parabiotic Rats.*

| | Pair No. | Sex of partners. | Duration of experiment (weeks). | Number of of doses of A.A.F. | Ovary. | Pituitary. |
|---|---|---|---|---|---|---|
| *Group I* | 1 | ♀/spayed ♀ | 18 | 18 | 0 | 0 |
| | 2 | Ditto | 16 | 18 | Gran. cell tum. | 0 |
| | 3 | „ | 19 | 18 | 0 | 0 |
| | 4 | „ | 25 | 20 | 0 | 0 |
| | 5 | „ | 20 | 18 | 0 | 0 |
| | 6 | „ | 27 | 17 | Gran. cell tum. | 0 |
| | 7 | „ | 38 | 17 | 0 | 0 |
| *Group II* | 1 | ♀/spayed ♀ | 19 | 10 | 0 | 0 |
| | 2 | Ditto | 41 | 14 | 0 | Adenoma |
| | 3 | „ | 36 | 21 | Carcinoma | „ |
| | 4 | „ | 26 | 24 | 0 | „ |
| | 5 | „ | 19 | 21 | Gran. cell tum. | 0 |
| | 6* | „ | 43 | 21 | „ „ | Adenoma |
| | 7 | „ | 36 | 21 | „ „ | „ |
| | 8 | „ | 19 | 21 | „ „ | 0 |
| | 9 | „ | 23 | 24 | „ „ | 0 |
| | 10 | „ | 20 | 25 | 0 | 0 |
| | 11 | „ | 25 | 24 | Gran. cell tum. | 0 |
| | 12 | „ | 28 | 24 | Gran.-theca tum. | 0 |
| | 13 | „ | 40 | 24 | 0 | Adenoma |
| *Group III* | 1 | ♀/castr.♂ | 16 | 19 | Gran. cell tum. | 0 |
| | 2 | Ditto | 24 | 17 | 0 | 0 |
| | 3 | „ | 35 | 20 | 0 | Adenoma |
| | 4 | „ | 21 | 20 | Gran. cell tum. | 0 |
| | 5 | „ | 31 | 20 | Gran. cell tum. (left). Theca cell tum. (right) | 0 |
| | 6 | „ | 38 | 20 | 0 | Adenoma |
| *Group IV* | 1 | ♀/spayed ♀ | 13 | 24 | Atypical proliferation of gran. | 0 |
| | 2 | Ditto | 15 | 24 | Gran. cell tum. (early) | 0 |
| | 3 | „ | 20 | 24 | Gran. cell tum. | 0 |

Groups I, II and III Wistar rats ; Group IV Piebald rats.
* Multiple mammary tumours.

macroscopically recognizable neoplasms. Fourteen tumours of the ovary were found in Wistar rats (Groups I, II and III), an incidence of over 50 per cent, and 2 in Group IV consisting of three pairs of piebald rats. In one Wistar rat both ovaries showed neoplastic changes, in the others only one gonad was tumourous, the left and the right side being affected with equal frequency. In all but 3 of the ovarian tumours granulosa cells were the prevailing cell type. Macroscopically they were of whitish colour, showing frequently areas of haemorrhagic discoloration. The whitish parts corresponded to solid, the reddish-brown ones to cystic blood-filled structures. The earliest neoplasms seen were generally round in shape and had a diameter of 3 to 4 mm. They were found in Wistar rats of Groups I to III after 16 to 20 weeks of parabiosis ; in piebalds they occurred even earlier. The largest granulosa cell tumour which had destroyed completely the ovary in which it originated measured 27 $\times$ 16 $\times$ 25 mm. and weighed 3·88 g. It was the only neoplasm of this kind to produce a metastasis, the secondary being situated in the adhesions linking the primary to neighbouring organs.

The material at our disposal allows the study of the development of granulosa cell tumours from their early stages. Fig. 2 shows a large haemorrhagic cyst found in an ovary of Rat 1, Group IV, sacrificed at the 13th week of the experiment. This cyst had a thin wall formed by fibrous connective tissue which in most places was poor in cells. In some areas the capsule was thickened, and here signs of previous haemorrhage such as pigment-loaded macrophages were present. Into the cavity papillary projections protruded. They were of two kinds, some with a narrow and others with a broad base. Into the former, delicate strands of spindle-shaped cells entered, forming their core. Into the latter, capillaries penetrated surrounded by cells having ample eosinophilic cytoplasm, and a nucleous less rich in chromatin than the cells which covered and formed the bulk of the projections. These had a nucleus rich in chromatin and a scanty cytoplasm. They were typical granulosa cells, while the ones having ample cytoplasm resembled lutein cells. This was the earliest neopalstic lesion found in our material, and shows that atypical growths of this kind were not necessarily permanent ones. They may become firmly established and fill the whole of the cystic cavity or they may be destroyed by haemorrhages. We have seen frequently in ovaries obtained from rats treated with A.A.F. and killed after more prolonged parabiosis haemorrhagic cysts, where only small nests of granulosa cells remained embedded in the dense connective tissue of the capsule while the cavity contained only inspissated blood and tissue debris. Such chocolate cyst-like lesions were absent from the ovaries of the control pairs not treated with A.A.F. Another animal of the same group sacrificed 2 weeks later was found to have in the ovary a macroscopically visible nodule formed almost entirely by granulosa cells. It was of about the same size as the early granulosa cell tumuor depicted in Fig. 3, 4 and 5, which it resembled closely. This tumour was found in an A.A.F.-treated albino rat after 19 weeks of parabiosis (Group II, Rat 5). Fig. 3 shows a sharply limited spherical nodule situated in an ovary containing cystic follicles. It was surrounded by a thin fibrous capsule. Between the capsule and the neighbouring cystic follicles were large blood-filled sinuses. Into the nodule entered capillaries accompanied by cells which either resembled theca or lutein cells as illustrated in Fig. 4. Fig. 5 shows the appearance of the granulosa cells forming the bulk of the nodule, and the tendency towards foci of liquefaction leading to the formation of cystic spaces within the tumour. The remaining rat of Group IV (Rat 3) was killed at the 20th week of the experiment. Both its ovaries were grossly enlarged ; the left weighing 600 g. is depicted in Fig. 6. A large whitish cyst forms the larger part of the gland. To the left of the cyst follows a white nodule having a few dark spots and the extreme left is occupied by a dark blood-filled cyst. A section from the region of the white nodule shows two distinct solid structures, one nearly filling a cavity, while the other occupies only a fraction of a neighbouring cystic follicle (Fig. 7). Histologically both nodules were composed almost entirely of granulosa cells with an admixture of luteinized cells. As in the earlier tumours described above, small cystic spaces were present within the agglomerations of granulosa cells (Fig. 8). At first glance it seemed that some of these cavities were surrounded by tall cylindrical cells with a basically situated nucleus. Closer inspection revealed that in reality the layer nearest the cavity was formed by ghost cells. Their nucleus was hardly stained by haematoxylin, so that these elements by their close contact with adjacent healthy granulosa cells simulated cylindrical cells. A section of the largest granulosa cell tumour found in Rat 5, Group III, is shown in Fig. 9. This tumour had essentially the same structure as the smaller early neoplasm of this kind. It contained large cystic areas besides solid areas formed by granulosa cells arranged in folliculoid pattern or in solid agglomerations (Fig. 10).

Only 3 tumours need to be described in greater detail. In Rat 12, Group II, the left ovary was greatly enlarged and its lateral half transformed into a hard tumour of whitish colour. Little ovarian tissue, apparently free of corpora lutea, was recognizable on naked-eye inspection. The organ weighed 286 mg., and was found to consist of areas of granulosa cells, nests of lutein cells and regions where theca-like cells predominated (Fig. 11). The tumorous right ovary of Rat 5, Group III, showed histologically an unusual picture. This ovary was also considerably enlarged (802 mg.) and fairly solid with only a few blood-filled cysts near its surface. Only a small rim of ovarian tissue remained ; most of the specimen was obviously neoplastic. Histologically this tumour was composed mainly of spindle-shaped cells with scanty cytoplasm, and a nucleus which varied considerably from elongated dense to oval or spherical vesicular. Among them small groups of cells were present having ample cytoplasm which stained well with eosin. These elements had a larger vesicular nucleus with the chromatin displaced towards the nuclear membrane. They were arranged in tubular structures, the lumen being filled with colloid-like material. Both types had well-defined cell borders. The most prominent features of this tumour were glomerulus-like structures formed by the spindle cells (Fig. 12). Tufts of these elements grew into cystic spaces which rarely showed a typical lining. Rarely one gained the impression that cells resembling flat epithelium lined the cavity surrounding the tuft (Fig. 13). Only the largest of these structures contained a blood-vessel, which entered the tuft together with the spindle cells by a narrow pedicle. Finally in some areas of the tumour the appearance and arrangement of cells resembled closely that seen in the granulosa cell tumour of the other ovary. We have classified this neoplasm as a theca cell tumour because the granulosa contributed only little to its make-up.

Another unusual neoplasm was found in Rat 3, Group II. In the 36th week of the experiment the health of the intact partner suddenly declined and therefore the pair was sacrificed. In the region of the left ovary there was a large blackish cyst, on the posterior wall of which some ovarian tissue was recognizable. The anterior surface showed nodular whitish-grey tissue covering part of the cyst. Many adhesions connected the cyst with the omentum and the colon, from which it could not be severed. Multiple small reddish cysts of about 1 mm. in diameter were found in the omentum and other parts of the abdomen and the lungs were riddled with nodules raised above the surface. These were of greyish colour or showed widespread haemorrhagic discoloration. Histological examination of the black cyst revealed that only in its periphery, i.e., near the fibrous capsule, cellular elements were present. These were of two kinds : tumour cells and cells of the granulation tissue which invaded from the periphery the inspissated blood and amorphous material filling the cyst. Two types of neoplastic cells could be distinguished : bizarre cells of unusually large size having one or, rarely, several nuclei with deeply stained prominent nucleoli. Their cytoplasm was ample and stained only weakly with eosin. Intermingled with these elements were nests of much smaller cells of epithelial character. These were polyhedral in shape and had well-marked cell borders, in contrast to the large elements which tended to form synyctia. The nuclei of the small cells were rich in chromatin and their cytoplasm was basophilic. The section reproduced in Fig. 14 was taken from the region where the tumour approached the colon. Here no invasion of the intestine is seen ; however, in other preparations it was found that the tumour cells had broken through the muscularis and invaded the submucosa. Widespread haemorrhages and lack of a stroma and of blood vessels were other characteristic features of this cancer. Fundamentally the same changes were seen in the lung. The larger the nodule the more prevailed haemorrhagic necrosis. Only the smallest deposits were free of haemorrhage, and consisted exclusively

EXPLANATION OF PLATES.

FIG. 1.—Intact parabiont with pyometra. × 0·3.
FIG. 2.—Haemorrhagic cyst with papillary ingrowths in hyperplastic ovary. × 12.
FIG. 3.—Early granulosa cell tumour. × 20.
FIG. 4.—Detail from Fig. 3 showing the capsule of the tumour, the blood supply, the arrangement of the granulosa cells and a nest of luteinized cells. × 65.
FIG. 5.—Detail from Fig. 3 showing the predominant cell type. × 330.
FIG. 6.—Hyperplastic ovary with clear and haemorrhagic cysts and early granulosa cell tumour. T indicates the area occupied by the tumour. (Pair 3, Group IV).
Fig. 7.—Section from tumour illustrated in Fig. 6 showing two distinct foci of tumour growth. × 13.
FIG. 8.—Detail from the larger nodule seen in Fig. 7. Granulosa cell tumour with admixture of lutein cells. × 90.
FIG. 9.—Section through the largest granulosa cell tumour observed. × 3.
FIG. 10.—Detail from tumour depicted in Fig. 9. × 65.
FIG. 11.—Area from a mixed granulosa-theca-lutein cell tumour. × 90.
FIG. 12.—Area from theca cell tumour showing “ glomerulus-like ” structures. × 110.
FIG. 13.—Another area of the theca cell tumour. × 330.
FIG. 14.—Area from anaplastic carcinoma showing the two types of tumour cells (L = large cell type, S = small cell type ; M = muscular coat of rectum). × 100.
FIG. 15.—Lung metastasis of tumour seen in Fig. 14. × 100.
FIG. 16.—Nest of atypical cells found in the ovary of intact partner (Pair 8, Group V). × 100.
FIG. 17.—Two small nodules found in the pituitary of the intact partner (Pair 13, Group II). × 65.
FIG. 18.—Detail from one of the nodules seen in Fig. 17 showing the transitions from fully granulated acidophils with large Golgi body to degranulated cells with similar Golgi apparatus. × 400.
FIG. 19.—Area from the other nodule seen in Fig. 17. Number of acidophils greatly reduced (appearing black in the microphotograph). Most cells have a large Golgi apparatus. × 240.
FIG. 20.—Section from fibroadenoma showing many secreting acini. × 60.
Fig. 21.—Atrophic uterus of the spayed partner of Pair 8, Group V. × 25.
FIG. 22.—Cystic hyperplastic breast gland of spayed partner of Pair 8, Group V. × 25.
FIG. 23.—Cortex of suprerenal of spayed partner of Pair 3, Group IV. Zona glomerulosa and outer fascicularis containing less lipoid than normally. (Oil Red O preparation.) × 60.
FIG. 24.—Cortex of suprarenal of a spayed rat. Glomerulosa loaded with fat droplets. (Oil Red O preparation.) × 60.

1
2
3
4
5
T
M/M
6

7

8

9

10

11

12

Bielschowsky and Hall.

13

14

15

16

17

18

Bielschowsky and Hall.

19

20

21

22

23

24

of tumour cells as depicted in Fig. 15. Mitoses were as frequent in the primary tumour as in the secondaries. A thorough search did not reveal the presence of teratomatous structures in the primary, nor were we able to trace with absolute certainty the origin of the neoplasm to the ovary since the oviduct was no longer recognizable. Many features of this cancer resembled those of chorion-epithelioma.

As already mentioned, no tumours of macroscopic size were found in the ovaries of the intact parabionts not treated with A.A.F. (Groups V, VI and VII). The only lesion suspicious of atypical growth was found in one of the ovaries of an intact female which survived parabiosis for 42 weeks (Group V, Rat 8). An irregularly shaped but sharply limited nest of cells with basophilic cytoplasm and nuclei fairly rich in chromatin was seen situated near the surface of the ovary (Fig. 16). Serial sections revealed the presence of one to two mitoses per section, but an ovum was not found. We are unable to state with certainty that this lesion was of neoplastic nature.

As already mentioned, no ovarian tumours were found in the 10 " single " albino rats which received 24 doses of 4 mg. of A.A.F. by stomach-tube (Group VIII). Among many hundred female rats treated with this carcinogen we have found only one instance of a neoplastic lesion in the female gonads. This was discovered in a rat $17\frac{1}{2}$ months of age when sacrificed. The rat had received 5 doses of A.A.F. by stomach-tube when 2 months old ; then the animal was kept without further treatment for 1 year, and for the last 16 weeks of her life she received methyl-thiouracil in the drinking water. At the post-mortem the left ovary was found to be converted into a large cyst measuring $20 \times 15 \times 10$ mm. On histological examination septa subdividing the cyst in several compartments and covered with granulosa cells were seen. Although macroscopically this cyst resembled the clear cysts which we have frequently found in the ovaries of parabiotic females and also in rats treated with stilboestrol, we have never observed in such structures proliferation of granulosa cells except in this one instance.

*The morphological signs of the endocrine imbalance existing in the intact partner.*

All signs of hyperoestrinism were present in the intact partner (Zeckwer, 1944). The vagina was lined by hyperplastic squamous keratinizing epithelium. In the few instances where the uterus was not heavily infected the epithelium lining the cavity was tall cylindrical and areas of squamous metaplasia were also present, the wall of the organ being thickened. In the infected uteri most of the epithelium was destroyed. Where rests remained it was generally squamous and keratinizing. The breast glands were grossly hyperplastic, and in most animals macroscopically visible cysts filled with a milky fluid were seen. Histologically such glands showed a pronounced cystic hyperplasia of the ducts and periductal fibrosis. The hyperplasia was not limited to the ducts, but alveolar growth had also occurred in many instances. The epithelial cells lining the alveoli showed marked secretory activity. The thymus was atrophic, and the supra-renals enlarged in size due to a hyperplasia of the cortex.

The pituitaries of the intact partners were invariably larger than those of the gonadectomized litter-mates, and already after 15 to 17 weeks of parabiosis had increased to an average of 8 mg. per 100 g. body weight. The largest observed (Rat 6, Group III, and Rat 8, Group V) reached the extraordinary weight of 74·8 and 139·3 mg. respectively. Such pituitaries produced signs of increased intracranial pressure as ataxia and were an additional factor for the declining health in rats surviving parabiosis for more than 30 weeks. The pituitaries of the intact partners were mostly solid and of whitish colour and haemorrhagic areas were not conspicuous. While the majority of these glands showed a symmetrical enlargement of the anterior lobe, in females surviving for more than 25 weeks of the experiment the pituitaries assumed an ever-increasing irregular shape. At first small whitish or slightly yellowish nodules appeared which were single or multiple. Later on the normal shape of the gland became more and more obscured by irregular growths, which compressed but never invaded the brain. The larger the tumour the greater was the tendency for brownish discoloration. These lesions occurred in intact partners independently of whether A.A.F. was given or not (Tables I and II).

TABLE II.—*Adenomata of the Pituitary in Intact Female Partner of Parabiotic Rats not Treated with A.A.F.*

| | Pair No. | Sex of partners. | Duration of experiment (weeks). | Pituitary. |
|---|---|---|---|---|
| *Group V* | 1 | ♀/spayed ♀ | 17 | 0 |
| | 2 | Ditto | 20 | Adenoma |
| | 3 | ,, | 22 | 0 |
| | 4 | ,, | 32 | 0 |
| | 5 | ,, | 30 | Adenoma |
| | 6 | ,, | 18 | 0 |
| | 7 | ,, | 26 | 0 |
| | 8 | ,, | 42 | Adenoma |
| | 9* | ,, | 18 | 0 |
| *Group VI* | 1 | ♀/spayed ♀ | 23 | Adenoma |
| | 2 | Ditto | 32 | ,, |
| | 3 | ,, | 16 | 0 |
| | 4 | ,, | 26 | 0 |
| | 5 | ,, | 40 | Adenoma |
| *Group VII* | 1 | ♀/castr. ♂ | 25 | 0 |
| | 2 | Ditto | 21 | Adenoma |
| | 3 | ,, | 28 | ,, |

* All rats from Wistar strain except Pair 9, Group V (Piebald).

Microscopically the hyperplastic glands were free of basophils. The acidophils tended to be large ; they had a vesicular nucleus with a large nucleolus and a very prominent Golgi apparatus. The degree of degranulation varied considerably. The partially degranulated forms resembled closely a type of chromophobes characterized by an enlarged Golgi apparatus. These cells became increasingly numerous the longer the experiment lasted. The cells composing the early nodules of the pituitary differed little from those in the rest of the anterior lobe except for a reduction in the number of acidophils. Another feature of these foci of nodular hyperplasia was an increased number of mitoses. Fig. 17 shows two nodules separated by a slightly compressed strand of pituitary cells, among which acidophils are fairly numerous, and Fig. 18 an area of one of these nodules photographed at a high magnification. In the latter all transitions from deeply granulated acidophils to apparently completely degranulated cells are recognizable. Common to many of these elements and independent of the degree of acidophilic granulation was the peculiar Golgi apparatus which in Papanicolaou preparations appeared as a ring-like structure, the negative Golgi image, surrounding some slightly brownish-stained granular material. Fig. 19, taken from the other nodule seen in Fig. 17, shows that the majority of the cells composing this structure were degranulated with only a few acidophils remaining. In the more advanced adenomata of the pituitary more atypical cells appeared but fundamentally they did not differ from the earlier lesions. In our material we have rarely seen adenomata completely free of acidophils. One or the other fully granulated and more frequently partially granulated acidophils were nearly always present. To conclude, we consider the nodular structures to be adenomata derived from cells of the acidophil series.

*Tumours of other organs observed in intact partners and in " single " rats of Group VIII treated with A.A.F.*

In the rats of Group VIII three adenocarcinomata of the breast and one squamous keratinizing epithelioma of the external auditory meatus were found (Table III). Two of the cancers appeared after an interval of 51 to 52 weeks, i.e., 9 weeks after the pair surviving longest had been sacrificed. Only one of the intact partners treated with A.A.F. developed tumours of the breast (Rat 6, Group II). In the 35th week of the experiment a mass was felt in the region of the fifth left mammary gland. It was surgically removed, and was found to be a well-encapsulated tumour measuring 20 × 12 × 9 mm. On cutting, milky fluid escaped under pressure. Histologically it was a fibroadenoma, in which the epithelial elements were much more numerous than is common in such tumours except when occurring in pregnant or lactating females. The breast gland in which the fibroadenoma originated showed the usual cystic hyperplasia typical for the mammary gland of intact partners and the tumour epithelium the same secretory activity as the glandular epithelium of the surrounding tissue (Fig. 20). Eight weeks later 2 more breast tumours appeared in this rat ; one was situated in the region of the 6th left breast gland and the other in the region of the 4th.

TABLE III.—*Tumours Induced by 24 Doses of 4 mg. of A.A.F. in " Single " Female Rats of the Wistar Strain (Group VIII).*

| Rat No. | Duration of experiment (weeks). | Breast. |
|---|---|---|
| 1 | 52 | 0 |
| 2 | 52 | 0 |
| 3 | 51 | Adenocarcinoma |
| 4 | 21 | ,, |
| 5 | 52 | 0 |
| 6* | 52 | 0 |
| 7 | 52 | 0 |
| 8 | 28 | Adenocarcinoma |
| 9 | 52 | 0 |
| 10 | 52 | 0 |

* Small carcinoma of external auditory meatus.

The former was macroscopically and histologically very similar to the neoplasm just described. The other was a small adenocarcinoma of the breast which showed far less secretory activity than the fibroadenoma which had developed simultaneously.

Benign cystic cholangiomata were present in practically all rats treated with A.A.F., but in the intact partners they were generally larger and more widespread than in the " single " animals, in which they were limited to a few cysts in lobus caudatus and left lobe. No malignant tumours of the liver were seen in any of these rats. The livers of the gonadectomized partners were always free of neoplastic lesions and were of normal size while those of the intact litter-mates were enlarged.

*The spayed partner.*

The pituitaries of the gonadectomized litter-mates showed always a marked increase in basophils, many of which were of the signet-ring type, and these glands were indistinguishable from those of " single " ovariectomized rats. The uterus and vagina were just as atrophic as in " single " castrates, but in some instances the breast glands were quite different from what we expected to see in a spayed animal. Instead of a few ducts lined by low cuboid epithelium many cystic ducts were present. The contrast between the atrophic uterus (Fig. 21) and the cystic dilated ducts of the mammary gland (Fig. 22) was especially striking in the spayed partner of Rat 8, Group V. We are unable to state why such changes occurred only in isolated instances.

Another feature in which the spayed parabiont differed from a " single " ovariectomized female was the cytology of the suprarenals. As depicted in Fig. 23 the zona glomerulosa and the outermost part of the fascicularis of the spayed partner showed a greatly diminished amount of lipoids, which was most pronounced in animals killed after 15 to 25 weeks of parabiosis. Fig. 24 is included for comparison ; it shows the distribution of lipoids in the cortex of the suprarenal of a " single " rat spayed 6 months previously. Here the zona glomerulosa was loaded with lipoids, and the outer fascicularis contained more droplets than towards the centre of the gland.

### DISCUSSION.

Drips and Ford (1932), studying the effects of roentgen rays on the oestrous cycle and on the ovaries of the rat, were the first to observe neoplastic changes in the ovary as late sequels of irradiation. A few years later Furth and Butterworth (1936) described tumours of the ovary which occurred in old mice exposed to X-rays in their youth. A second experimental method of obtaining such neoplasms was discovered by Biskind and Biskind (1944), who obtained such tumours by transplanting ovaries into the spleen of gonadectomized rats. More recently Fels (1949) observed two ovarian tumours, one thecoma and one granulosa cell tumour, in rats subjected previously to a temporary ligature of the blood vessels entering the ovary. Finally, Moon, Simpson, Li and Evans (1950) described neoplastic lesions in the female gonads of rats treated with pure growth hormone for 1 to $1\frac{1}{2}$ years. All the methods quoted induce an endocrine imbalance. X-ray radiation of the body as well as irradiation of the ovaries leads to partial or complete castration, as indicated by a more or less pronounced disturbance of the oestrous cycle, which is temporary or permanent, depending on the dosage given.

Lick, Kirschbaum and Mixer (1949), as well as Kaplan (1950), have shown conclusively that intact ovarian endocrine function inhibits the development of ovarian tumours in irradiated mice. In other words ovarian tumours develop only in animals in which the normal pituitary-ovarian relationship is disturbed for some time after the exposure to X-rays. Levine and Witschi (1933) found that a normal female rat joined in parabiosis to an animal whose ovaries had been exposed to X-rays went into continuous oestrus. The gonads of the intact partners were composed of cystic follicles—proof of increased secretion of follicle-stimulating hormone (F.S.H.) by the pituitary of the irradiated parabiont.

The liver of rodents inactivates ovarian steroids so efficiently that the secretions of a gonad grafted into the spleen do not enter the general circulation. In consequence the pituitaries of animals bearing such grafts are of the castrate type, and have an elevated gonadotropin content, as shown by Greep and Jones (1950). Fels (1949) discovered that a temporary ligature of the blood vessels supplying the ovaries led to an endocrine imbalance characterized by hyperoestrinism in the presence of castration changes in the pituitary. It seems therefore that one factor is common to at least 3 of the 4 methods quoted above; they induce an endocrine imbalance characterized by pituitaries secreting elevated amounts of gonadotropins. It is therefore not astonishing that the neoplastic changes induced under these experimental conditions are all of the same kind. Nearly all growths so obtained belong to the group of granulosa-theca-lutein cell tumours. Moon, Simpson, Li and Evans (1950) also regard the neoplastic changes observed in the ovaries of rats treated with growth hormone to be due to a pituitary imbalance. These authors mention a reduction of the acidophils and an increase of the chromophobes in the pituitaries of such rats, and noted that in 2 animals the histological picture of the anterior pituitary was of the castration type. It seems therefore that the basophils and especially the F.S.H. secreting cells can increase in animals treated with growth hormone. However, more information is required before one can assume that ovarian neoplasms obtained by treatment with growth hormone are due to the same mechanism as the other experimental tumours of the ovary, i.e., to an excess of gonadotropins.

In our case there can be no doubt that tumour development in the female gonad was the result of increased stimulation by gonadotropins and of the carcinogenic action of A.A.F. It is especially the follicle-stimulating hormone secreted in elevated amounts by the gonadectomized partner which determines the endocrine situation. Admittedly during the first weeks of parabiosis there is good evidence for the action of luteinizing hormone, as indicated by the presence of many healthy corpora lutea in the ovary of the intact litter-mate. Later, when the increased secretion of oestrogens from the stimulated ovary has suppressed all the gonadotrophic activity of the intact partner's pituitary and continuous oestrus has become established, the pituitary hormone dominating the picture is F.S.H. It is therefore not astonishing that granulosa cell tumours are so frequent in our material and that luteinization occurred to only a limited extent. We believe we have found enough evidence to postulate that these neoplasms originate from what Drips and Ford (1932) call hyperplastic follicular structures. Fig. 4, 6 and 7 of their paper resemble closely the early lesions observed by us, and which we have seen to progress to tumours of considerable size. It seems unfortunate that Drips and Ford (1932) compared the atypical structures observed by them in the irradiated ovaries of rats with carcinomatous ovarian cystadenomata of the human. The undoubtedly decisive role of F.S.H. in the pathogenesis of the tumours described in this paper forces us to reject several of the hypotheses concerning the histogenesis of this class of ovarian neoplasms. There is general agreement that F.S.H. is responsible for the growth of follicles, but there is very little evidence that it is necessary for the formation of new follicles. The normal interplay between F.S.H. and luteinizing hormone (L.H.) in the ovarian cycle is greatly disturbed in the parabiotic female joined to a gonadectomized litter-mate. After 3 to 4 months of parabiosis the ripe follicle is not any more transformed into a corpus luteum nor does it involute, but is exposed to continued stimulation, which does not cease after the death of the ovum. This is in our opinion the decisive factor which leads to the development of the granulosa cell tumours. Contrary to what is seen in spleen grafted or irradiated ovaries of mice, in the rat there is not the slightest evidence that these tumours " arise from proliferation and ingrowth of germinal epithelium ", as assumed by Li and Gardner (1947). We have observed in some glands small tufts of cells growing out from the surface of the ovary, but we have never seen tubular ingrowths from " germinal epithelium ". Origin from embryonic nests seems a most unlikely hypothesis, since many workers obtained ovarian tumours in 50 to 70 per cent of their experimental animals (Kaplan, 1950; Furth and Sobel, 1948). Finally, origin from ovarian mesenchyme has to be considered. Undoubtedly the stroma cell of the ovary can differentiate into theca or granulosa cells, and therefore one cannot exclude this possibility. Still, we prefer origin from differentiated granulosa cells because of the morphology of the early lesions observed by us. They are frequently spherical, and surrounded by theca cells like normal follicles. In our opinion they arise from granulosa cells which survive the death of the ovum, the organization centre of the granulosa, and their point of origin are papillary ingrowths situated on the wall of cystic follicles. Furth and Butterworth (1936) also expressed the opinion that the granulosa cell tumours discovered by them in previously irradiated ovaries " may originate from remnants of disorganized follicles ".

That theca cells participated and in one instance became the main elements in the ovarian tumours obtained by us is in accordance with the hypothesis of follicular origin. If one tries to find an explanation for the " glomerulus-like " structures depicted in Fig. 12 and 13 one has not to fall back on the conception of disturbed embryogenesis. In preovulatory follicles invaginations of the theca accompanying tufts of capillaries are a common feature.

The chorion epithelioma-like tumour presented some diagnostic difficulties. We consider it to be an anaplastic carcinoma. As pointed out by Willis (1947), anaplastic carcinoma can simulate chorion epithelioma and therefore more than morphological similarity is required to establish the diagnosis, in the absence of pregnancy. The lack of teratomatous elements and the lack of signs of functional activity such as corpus luteum cysts in the unaffected right ovary are in favour of the diagnosis of anaplastic carcinoma. As far as the origin of this cancer is concerned we are inclined to assume that it arose in the left ovary, because we have observed more than once the presence of atypical cells of large size showing mitotic activity in the wall of haemorrhagic ovarian cysts. In addition it might be mentioned that Li, Gardner and Kaplan (1947) described small aggregates of giant cells in an experimental tumour of the ovary of a mouse. Also we have been unable to find references to tumours of the oviduct of rodents.

We have been impressed by certain similarities in the development of tumours of the thyroid induced by A.A.F. and prolonged treatment with a goitrogen and of the ovarian tumours which are the subject of this paper. In both cases a general hyperplasia of the glands is followed by a focal hyperplasia of a special kind which leads ultimately to malignant growth. If gonadotropines alone can induce ovarian neoplasms, as seems likely from the results of Biskind and Biskind (1944), one should expect them to appear also after long-continued parabiosis. So far we have been unable to maintain our pairs for more than 42 weeks, a period which seems too short to bring about this result without the aid of a carcinogen. To judge from the experience of Moon, Simpson, Li and Evans (1950) with growth hormone and of Griesbach, Kennedy and Purves (1945) with thyrotropic hormone, pituitary hormones appear to be slow acting carcinogenic agents. In both cases periods of more than a year were needed to obtain macroscopically recognizable tumours. However, tumour development in the thyroid was much more rapid, when A.A.F. was given simultaneously with or previously to the treatment with goitrogen.

Spontaneous ovarian tumours and especially granulosa cell tumours are exceedingly rare in rats (Iglesias, Sternberg and Segaloff, 1950), and therefore do not complicate the interpretation of the experiments reported in this paper. Finally an explanation has to be given why the incidence of granulosa cell tumours in Group I is lower than in Groups II and III. We believe that two factors are responsible ; lower dosage of A.A.F. and most probably also our surgical technique. Group I represents our first parabiotic pairs, in which we did not succeed as well as later on to obtain wide junctions, measuring 6 to 10 cm. at the time the animals were sacrificed.

The study of a number of organs of the intact partner showed clearly the effects of oestrogens secreted in excessive amounts by the stimulated ovaries. One of the sequels of this hyperoestrinism were the pituitary adenomata which occurred whether or not A.A.F. had been given. These neoplasms are indistinguishable from those obtained after long-continued treatment of rodents with large amounts of natural or synthetic oestrogens. Most authors describe them as chromophobe adenomata, and undoubtedly in advanced lesions only few granulated acidophilic cells are seen. We believe, however, that originally these benign neoplasms were acidophilic, just as in Zondek's case (1940). This author observed an adenomatous lesion composed of acidophils in the pituitary of a woman treated with very high doses of oestradiol.

Whereas the increased secretion of oestrogens by the stimulated ovaries led to neoplastic changes in the pituitary, no mammary tumours were observed in the control pairs, contrary to the findings of Zeckwer (1944), and only one of the intact parabionts treated with A.A.F. developed tumours of the breast gland. The material at our disposal does not allow the conclusion that the high level of oestrogen in the circulation of the intact partner inhibited the carcinogenic action of A.A.F. on the mammary gland ; but certainly this condition did not enhance it. On the other hand, the benign cystic cholangiomata of the liver were larger and far more widespread in the intact parabionts of Groups I to IV than in the "single" rats of Group VIII. This is in accordance with the findings of Stasney, Paschkis, Cantarow and Rothenberg (1947), who discovered that the administration of oestradiol " intensified the cytsic and hepatic lesions induced by A.A.F ".

SUMMARY.

(1) Benign and malignant tumours have been induced by A.A.F. in intact female rats joined in parabiosis to gonadectomized litter-mates. Ovarian tumours developed in about 50 per cent of these animals.

(2) A description of the histology of the ovarian neoplasms has been given and their histogenesis discussed. It has been found that most of them were of the granulosa cell type. F.S.H. secreted in excess by the pituitary of the gonadectomized litter-mate is regarded to be the factor essential for their development.

(3) The pituitary tumours observed in intact parabiotic females are considered to be due to the excess of oestrogens secreted by their stimulated ovaries. They occurred independently whether A.A.F. was given or not.

(4) The gonadectomized partners were always found to be free of neoplastic lesions, whether or not their litter-mates received A.A.F.

(5) The state of hyperoestrinism present in the intact parabiont did not enhance the carcinogenic action of A.A.F. on the mammary gland. When similar doses of A.A.F. were administered to "single" female rats two mammary cancers appeared during the first 40 weeks of the experiment, a period which corresponds to the maximum survival of our parabiotic pairs.

(6) Benign cystic cholangiomata were found in nearly all rats treated with A.A.F. In the intact parabionts they were more numerous and larger than in the "single" rats.

We wish to thank Professor R. A. Willis for giving us his opinion on the anaplastic carcinoma described in this paper, and Mr. E. R. Macdonald for photographing the specimen depicted in Fig. 1, 6 and 9.

REFERENCES.

BIELSCHOWSKY, F., AND HALL, W. H.—(1951) *Brit. J. Cancer*, **5**, 106.

BISKIND, M. S., AND BISKIND, G. T.—(1944) *Proc. Soc. exp. Biol., N.Y.*, **55**, 176.

DRIPS, D. G., AND FORD, F. A.—(1932) *Surg. Gynec. Obstet.*, **55**, 596.

FELS, E.—(1949) *Obstet. Ginec. Lat.-Amer.*, No. 9, 439.

FURTH, J., AND BUTTERWORTH, J. S.—(1936) *Amer. J. Cancer*, **28**, 66.

*Idem* AND SOBEL, H.—(1948) *J. nat. Cancer Inst.*, **8**, 7.

GREEP, R. O., AND JONES, I. C.—(1950) *Recent Progress in Hormone Research*, **5**, 197.

GRIESBACH, W. E., KENNEDY, T. H., AND PURVES, H. D.—(1945) *Brit. J. exp. Path.*, **26**, 18.

IGLESIAS, K., STERNBERG, W. H., AND SEGALOFF, A.—(1950) *Cancer Res.*, **10**, 668.

JACOBSOHN, D.—(1948) *Acta physiol. scand.*, **17**, suppl., 57.

KAPLAN, H. S.—(1950) *J. nat. Cancer Inst.*, **11**, 125.

LEVINE, W. T., AND WITSCHI, E.—(1933) *Proc. Soc. exp. Biol., N.Y.*, **30**, 1152.

LI, M. H., AND GARDNER, W. U.—(1947) *Cancer Res.*, **7**, 549.

*Iidem* AND KAPLAN, H. S.—(1947) *J. nat. Cancer Inst.*, **8**, 91.

LICK, L., KIRSCHBAUM, A., AND MIXER, H.—(1949) *Cancer Res.*, **9**, 532.

LILLIE, R. D.—(1948) 'Histopathologic Technic.' Philadelphia (Blakiston).

MOON, H. D., SIMPSON, M. E., LI, CH. H., and EVANS, H. M.—(1950) *Cancer Res.*, **10**, 549.

STASNEY, J., PASCHKIS, K. E., CANTAROW, A., AND ROTHENBERG, M. S.—(1947) *Ibid.*, **7**, 356.

WILLIS, R. A.—(1948) 'Pathology of Tumours.' London (Butterworth).

ZECKWER, I. T.—(1944) *Arch. Path.*, **38**, 99.

ZONDEK, B.—(1940) *J. Amer. med. Ass.*, **114**, 1850.